# Pulmonary Hydatid Cyst Mimicking Both Tuberculous Cavity and Aspergilloma: A Diagnostic Challenge in an Endemic Region

**DOI:** 10.1002/ccr3.71026

**Published:** 2025-09-29

**Authors:** Mohammad Alaa Aldakak, Aows Ahmad, Raneem Ahmad, Bassel Ibrahim, Yehia Taifour

**Affiliations:** ^1^ Faculty of Medicine Damascus University Damascus Syrian Arab Republic; ^2^ Faculty of Medicine Al‐Mouwasat University Hospital, Damascus University Damascus Syrian Arab Republic

**Keywords:** case report, *Echinococcus granulosus*, pulmonary hydatid cyst, tuberculosis mimic

## Abstract

Cystic echinococcosis (CE), caused by *Echinococcus granulosus*, is a zoonotic disease endemic in many pastoral regions. Pulmonary involvement, although less common than hepatic, may present with nonspecific respiratory symptoms and mimic other pathologies such as tuberculosis, posing a diagnostic challenge. We report a case of a 17‐year‐old Arab female from an endemic area who presented with cough, hemoptysis, fever, and general malaise. Chest imaging revealed a cavitary lesion in the upper lobe, raising suspicion of pulmonary tuberculosis or fungal infection. Laboratory findings showed leukocytosis and elevated inflammatory markers. Bronchoscopy and microbiological workup were negative for TB and fungi, though *Klebsiella* was cultured. Despite initial improvement with antibiotics, the definitive diagnosis was established intraoperatively after surgical resection revealed a complicated hydatid cyst. Postoperative culture identified *Pseudomonas*, and histopathology confirmed hydatid disease. This case highlights the diagnostic difficulty of pulmonary CE, particularly when imaging mimics tuberculosis. The upper lobe location and cavitary appearance contributed to initial misdirection. A lack of response to empirical therapy should prompt reconsideration of differential diagnoses, especially in endemic regions. Pulmonary hydatid cysts may present atypically and resemble more prevalent conditions like tuberculosis. Clinical vigilance, especially in endemic areas, is critical for timely diagnosis and appropriate management.

**Summary**

- In endemic areas, pulmonary hydatid cysts may mimic tuberculosis both clinically and radiologically.
- Lack of response to empirical treatment should prompt reconsideration of differential diagnoses.
- Early surgical intervention combined with antiparasitic therapy is essential for definitive diagnosis and favorable outcomes.

## 1 Introduction

Cystic echinococcosis (CE), or hydatid disease, is a zoonotic parasitosis caused by *Echinococcus granulosus* and classified by the World Health Organization as a neglected tropical disease. It is endemic in pastoral regions—particularly in the Mediterranean, Middle East, Central Asia, and South America—with prevalence rates in community surveys of 5%–10% and hospital‐based incidence reaching approximately 50 per 100,000; pulmonary involvement occurs in 20% of cases [1, 2]. Clinical manifestations depend on cyst location and complications. In pulmonary CE, early stages may be asymptomatic, but enlarged, ruptured, or infected cysts often present with cough, chest pain, dyspnea, or hemoptysis—symptoms that overlap with pulmonary tuberculosis or abscess and may mislead diagnosis [3]. Diagnosis primarily relies on imaging. Chest radiographs typically reveal well‐defined cystic opacities, while computed tomography scans (CT scans) offer detailed visualization of cyst walls, internal membranes, and features of rupture or secondary infection [4]. Serological tests such as Enzyme‐Linked Immunosorbent Assay (ELISA) and indirect hemagglutination serve as adjuncts but are less sensitive for pulmonary cysts [5]. Therapeutic strategies vary by cyst complexity. Small, uncomplicated cysts can be managed with benzimidazole therapy, particularly albendazole. However, symptomatic, large, or complicated cysts require surgical intervention. Combining perioperative albendazole with surgery significantly reduces recurrence rates [6]. Postoperative albendazole (10–15 mg/kg/day for ≥ 2 months) is advised for complicated or multiple cysts [7]. This rare illness, known as alveolar hydatid, presents with unfamiliar clinical signs and nonspecific radiological characteristics. Additionally, the disorder is associated with notable morbidity, warranting a strong index of suspicion and the implementation of further imaging studies to enable prompt intervention and avert the development of subsequent complications arising from the disease [8].

## 2 Case Presentation

### 2.1 Case History/Examination

A 17‐year‐old Arab female patient, a high school student residing in an urban area, nulliparous, non‐smoker, and non‐alcoholic, presented to the pulmonary clinic with a chief complaint of hemoptysis. Upon detailed history‐taking, she reported a dry cough producing yellowish sputum, accompanied by a single episode of moderate hemoptysis that occurred 5 months ago. This was associated with fatigue, general malaise, decreased appetite, and recurrent daily febrile peaks responsive to oral antipyretics. She denied any associated chest pain, dyspnea, or night sweats.

The patient's past medical history revealed a documented allergic reaction to levofloxacin. There were no other significant medical, surgical, pharmacological, or familial antecedents. Systemic review and physical examination were largely normal, except for fine crackles in the upper half of the left lung field.

### 2.2 Clinical Examination

Vital signs were as follows: blood pressure 120/70 mmHg, heart rate 94 beats per minute, body temperature 38.5°C, and oxygen saturation (SpO_2_) 95% on room air. Laboratory investigations showed leukocytosis with a white blood cell count of 16,000/mm^3^, predominantly neutrophilic (*N* = 90%), and an elevated C‐reactive protein level (CRP = 60 mg/L).

## 3 Differential Diagnosis, Investigations and Treatment

**FIGURE 1:**
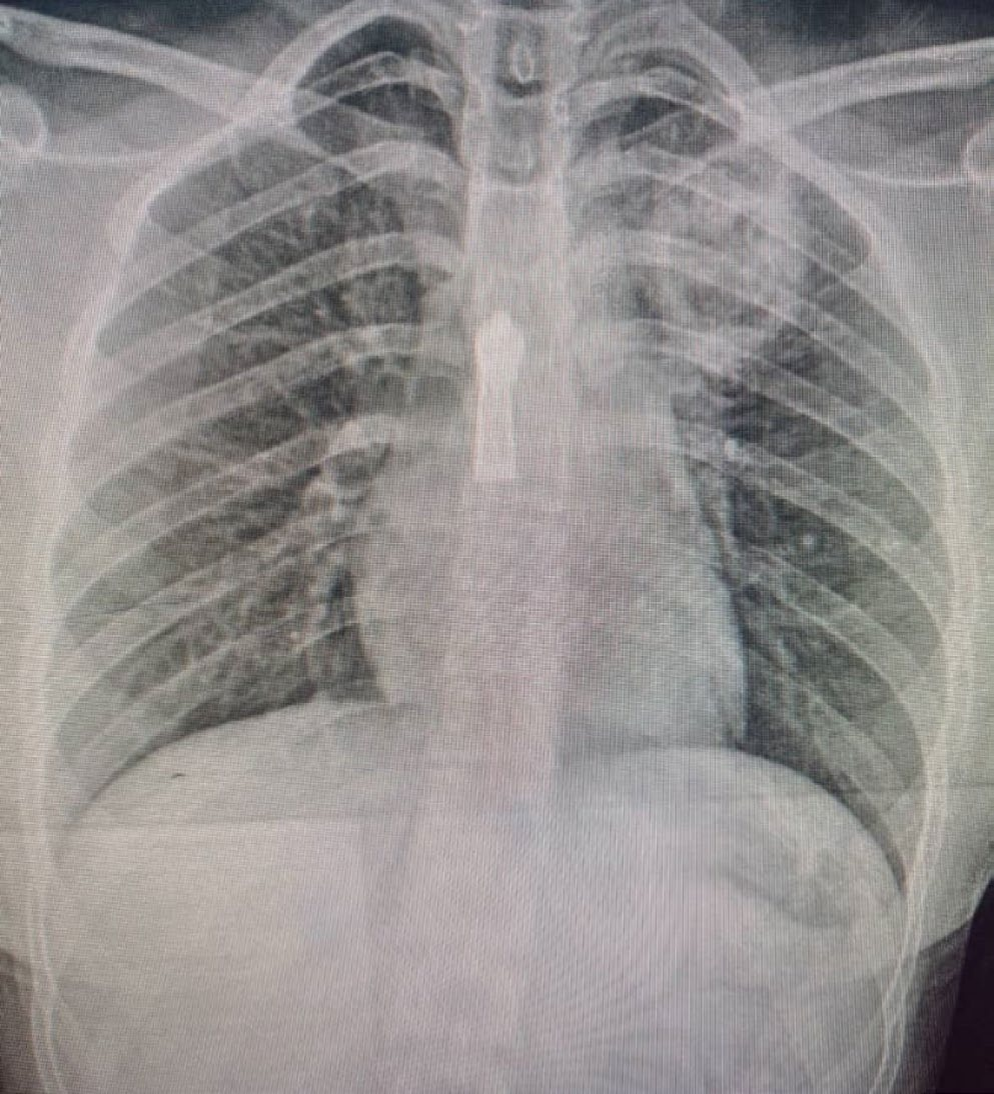
Chest X‐ray showing a cavitary opacity in the apex of the left lung, initially suggestive of a tuberculous cavity.

**FIGURE 2:**
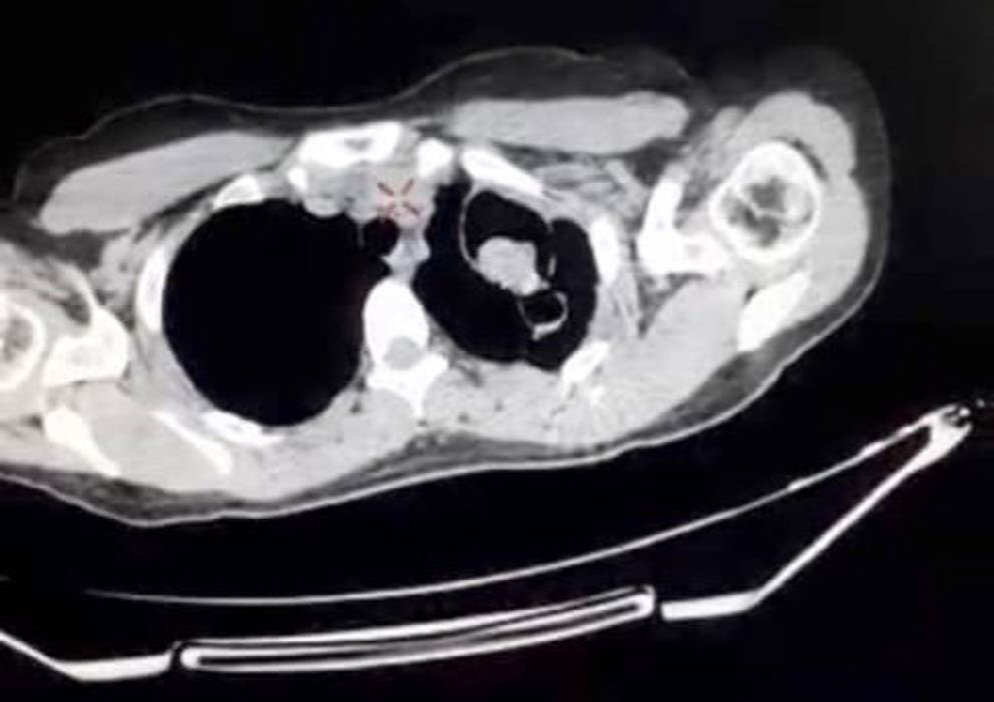
Axial chest CT scan (mediastinal window) revealing a well‐defined cavitary lesion in the left upper lobe with an internal round structure resembling a fungal ball.

**FIGURE 3:**
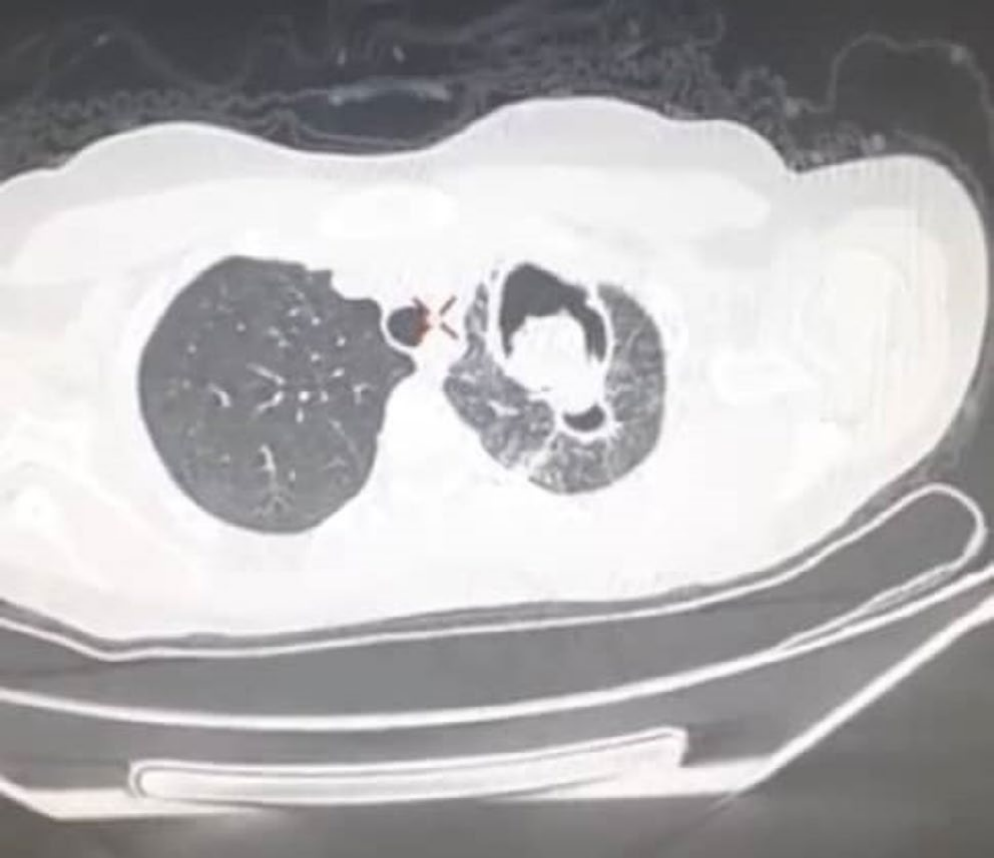
Axial chest CT scan (lung window) confirming the presence of an air‐fluid level within the left upper lobe cavity, suggestive of cyst rupture or secondary infection.

A chest X‐ray revealed a cavitary lesion in the apex of the left lung (Figure 1), followed by a CT scan showing a pulmonary cavity in the left upper lobe with a rounded, bell‐shaped intracavitary structure resembling a fungal ball (Figures 2 and 3).

Based on the radiological findings, the patient underwent bronchoscopy, which was reported as normal. Bronchial lavage was performed, and samples were sent for bacterial culture and sensitivity testing, *Mycobacterium tuberculosis* Polymerase Chain Reaction (PCR), mycobacterial culture and drug sensitivity testing, and fungal analysis. Results were negative for both mycobacterial and fungal studies. However, the bacterial culture yielded Klebsiella species sensitive to ceftriaxone, imipenem, gentamicin, and amikacin. Following the results of culture and sensitivity testing, the patient was started on a 2‐week course of ceftriaxone.

Based on the persistent radiological findings and incomplete resolution despite appropriate antibiotic therapy, surgical intervention was indicated due to the complexity and persistence of the lesion, suspected rupture, and the risk of further complications. A left thoracotomy was performed, which revealed a complicated hydatid cyst in the upper lobe of the left lung. The cyst was opened and drained, with excision of the laminated membrane, followed by irrigation and debridement of the residual cavity. The cavity was then sutured using the capitonnage technique, and a chest tube was placed.

Postoperative cultures of the cyst fluid identified Pseudomonas spp. sensitive to Ciprofloxacin, Rocephin, Tienam, Doxycycline, and Meropenem. Histopathology confirmed the diagnosis of hydatid disease.

## 4 Conclusion and Results (Outcome and Follow‐Up)

The chest drain was removed 3 days postoperatively, and a follow‐up chest X‐ray was obtained. The patient was started on Albendazole therapy (400 mg orally, twice daily) for 6 months to prevent recurrence and manage any potential residual disease. She showed progressive clinical and radiological improvement, with no signs of recurrence or complications on follow‐up.

## 5 Discussion

Hydatid disease, caused by *E. granulosus*, is endemic in regions where sheep farming is common and close contact with infected dogs is frequent [9]. While the liver is the most commonly affected organ (≈70%), pulmonary involvement occurs in up to 30% of cases [10, 11]. Infection begins with ingestion of parasite eggs, which hatch in the gastrointestinal tract and migrate via the portal or lymphatic systems to the lungs, where cysts develop [11, 12, 13]. In children, cysts grow faster and more frequently than in adults, possibly due to close contact with pets, often leading to growth delay [12, 13]. Males are diagnosed more often, possibly due to occupational exposures [12]. Pulmonary cysts can remain silent for 5–20 years before becoming symptomatic [11, 12]. However, early‐stage hydatid cysts are typically asymptomatic [12]. As they gradually enlarge—at rates ranging from 1 to 50 mm per year—they may exert pressure on adjacent structures, rupture, or become secondarily infected. The resulting clinical manifestations vary depending on the organ involved: hepatic cysts often present with abdominal discomfort and anorexia, whereas pulmonary cysts more commonly lead to cough, chest pain, and hemoptysis [9]. In cases of cyst rupture, patients may develop systemic symptoms such as fever, urticaria, eosinophilia, or even anaphylactic reactions. While the lower lobes are most frequently affected in pulmonary involvement [12], rupture occurs in nearly 50% of cases, with approximately 4% progressing to pneumothorax [14]. Triggers for rupture include thoracic trauma, forceful coughing, or repeated sneezing episodes [12]. Although multiorgan hydatid disease is uncommon, it typically results from silent or traumatic rupture with secondary dissemination [15]. Given these overlapping symptoms, researchers emphasize that *E. granulosus*, *M. tuberculosis* , and Lophomonas blattarum should all be considered in the differential diagnosis—particularly in patients presenting with eosinophilia, severe or persistent respiratory infections, underlying immunosuppression, or failure to respond to empirical antimicrobial therapy [16]. Our patient's presentation aligns with the typical epidemiological and clinical features of pulmonary hydatid disease. Living in an endemic area and presenting with cough, hemoptysis, and fever, along with a cavitary upper lobe lesion, initially mimicked tuberculosis. The absence of microbiological evidence and failure to respond to antibiotics led to surgical confirmation of a complicated hydatid cyst, reflecting the diagnostic challenges highlighted in the literature.

Diagnosis relies on clinical suspicion, imaging, and laboratory tests. Auscultation may reveal dullness, but findings are non‐specific. Chronic fever and weight loss can mimic malignancy. Rarely, patients may cough up grape‐skin–like cyst material, a pathognomonic but uncommon sign [12]. Lab tests may reveal eosinophilia and elevated IgE, though the latter lacks specificity in endemic regions [11, 12]. Imaging plays a central role. Chest X‐ray may reveal smooth, round opacities; CT offers better characterization, especially of ruptures and air‐fluid levels [14, 17, 18]. Ultrasound helps stage cysts as active, transitional, or inactive—guiding treatment decisions. Daughter cysts with a honeycomb appearance on ultrasound are diagnostic. Differential diagnoses include abscess, AV malformation, malignancy, or metastases [12]. In our case, the patient presented with respiratory symptoms including cough, hemoptysis, and recurrent fever—clinical features that closely align with the symptomatic stage of pulmonary hydatid disease. Radiologically, the cyst appeared as a cavitary lesion in the upper lobe, closely resembling a tuberculous cavity. In addition, CT demonstrated an intracavitary structure mimicking a fungal ball, which closely resembles aspergilloma. This radiological similarity has been previously reported and may contribute to diagnostic misdirection. The atypical location and imaging findings contributed to early confusion with more common pulmonary conditions. Moreover, the lack of clinical improvement following empirical antibiotic therapy highlighted the need to broaden the differential diagnosis.

Management of hydatid disease includes antiparasitic therapy (e.g., albendazole), Puncture, Aspiration, Injection, Re‐aspiration (PAIR), and surgery. While PAIR offers a minimally invasive alternative and shows comparable efficacy over 2 years [10], surgical excision remains the gold standard for large, ruptured, or complicated cysts due to its ability to prevent intrapulmonary spread [10, 13, 19]. Preoperative albendazole reduces intracystic pressure and facilitates safer removal, while postoperative administration lowers recurrence risk. Without treatment, mortality may reach up to two‐thirds, though modern therapy reduces it to approximately 3% [12].

## 6 Conclusion

Pulmonary hydatid disease remains a diagnostic challenge, particularly when it mimics more common conditions such as tuberculosis. In endemic regions, clinicians should maintain a high index of suspicion when confronted with cavitary lung lesions that fail to respond to conventional therapy. This case underscores the importance of integrating clinical, radiological, and surgical findings to establish a definitive diagnosis. Early recognition and appropriate surgical management, supported by antiparasitic therapy, are essential to prevent complications and ensure favorable outcomes.

## Author Contributions

**Mohammad Alaa Aldakak:** writing – original draft, writing – review and editing. **Aows Ahmad:** resources, writing – original draft. **Raneem Ahmad:** supervision. **Bassel Ibrahim:** supervision. **Yehia Taifour:** supervision, writing – review and editing.

## Ethics Statement

Institutional Review Board (IRB) approval is not required for de‐identified single case reports or case histories, in accordance with institutional policies.

## Consent

Written informed consent was obtained from the patient for publication and any accompanying images. A copy of the written consent is available for review by the Editor‐in‐Chief of this journal on request.

## Conflicts of Interest

The authors declare no conflicts of interest.

## Data Availability

Data available on request from the authors.
